# Gallic acid ameliorates dextran sulfate sodium-induced ulcerative colitis in mice *via* inhibiting NLRP3 inflammasome

**DOI:** 10.3389/fphar.2023.1095721

**Published:** 2023-01-25

**Authors:** Tian-Yuan Yu, Yi-Ming Feng, Wei-Song Kong, Shan-Ni Li, Xue-Jiao Sun, Gui Zhou, Rui-Fang Xie, Xin Zhou

**Affiliations:** ^1^ Department of Pharmacy, Longhua Hospital, Shanghai University of Traditional Chinese Medicine, Shanghai, China; ^2^ Institute of Chinese Medical Sciences, University of Macau, Macau, China; ^3^ Department of Pharmacy, Shanghai Municipal Hospital of Traditional Chinese Medicine, Shanghai University of Traditional Chinese Medicine, Shanghai, China; ^4^ Shanghai Nanyang Model Private High School, Shanghai, China; ^5^ Fengdu County People’s Hospital of Chongqing, Chongqing, China

**Keywords:** Gallic acid, ulcerative colitis, NLRP3, dextran sulfate sodium, Qingchang Suppository

## Abstract

**Background:** Ulcerative colitis (UC) is a chronic recurrent inflammatory bowel disease (IBD). The conventional drugs for UC may induce severe side effects. Herbal medicine is considered as a complementary and alternative choice for UC.

**Purpose:** This study aims to estimate the effect of natural polyphenol gallic acid (GA) on the NLRP3 inflammasome with dextran sulfate sodium (DSS)-induced colitis in mice.

**Study design:** The body weights and symptoms of BALB/c mice were recorded. Histological evaluation, ELISA, q-PCR, immunohistochemistry, and western blotting were carried out to observe the morphology, cytokine contents, mRNA expressions, and protein expressions, respectively. Lipopolysaccharide (LPS)-induced RAW264.7 macrophage was used to probe GA’s effect on relative protein expression.

**Results:** GA attenuated weight loss (*p* < 0.05), relieved symptoms, and ameliorated colonic morphological injury (*p* < 0.05) in mice with colitis induced by DSS. GA also lowered the contents of TNF-α, IL-1β, IL-18, IL-33, and IFN-γ in the serum and colon of mice, which were elevated by DSS, downregulated protein, and mRNA expressions of the NLRP3 pathway in the colon tissue. Furthermore, GA downregulated the expressions of NLRP3 (*p* < 0.05), iNOS (*p* < 0.01), COX2 (*p* < 0.01), and P-p65 (*p* < 0.05), and suppressed NO release (*p* < 0.001) in LPS-induced RAW264.7 cells.

**Conclusion:** GA ameliorated DSS-induced UC in mice *via* inhibiting the NLRP3 inflammasome. These findings furnish evidence for the anti-inflammatory effect of herbal medicines containing GA on UC.

## Introduction

Ulcerative colitis (UC) is a chronic relapsing inflammatory bowel disease (IBD) (Conrad et al., 2014). Long-term UC is also associated with a defined risk of colorectal cancer (Yashiro, 2014; Kvorjak et al., 2020). Nowadays, UC is generally believed to be connected to genetic, environmental, and immunological factors and is regarded as a multi-factorial disease. Aminosalicylates, immunosuppressants, steroids, and biological agents are the main options for UC treatment (Ungaro et al., 2017). However, their clinical applications have been greatly impeded by various side effects. Hence, there is an urgent need to find some other alternatives for UC treatment.

Recently, herbs and dietary supplements such as cannabis and curcumin have been considered as possible approaches to IBD (Cheifetz et al., 2017). Gallic acid (GA) is a polyphenol which is broadly distributed in tannin-producing plants such as tea, cocoa, and walnuts (Roche et al., 2017). As a natural compound, GA was reported to possess anti-toxic (Gomes et al., 2020), cardio-protective (Yan et al., 2019), and, especially, anti-inflammatory properties (Pandurangan et al., 2015). Previous reports showed that Qingchang Suppository (QCS), a herbal preparation, ameliorated colonic vascular permeability and exhibited an anti-inflammation effect on experimental colitis *in vivo* (Sun et al., 2018; Zhou et al., 2021), while GA, the main ingredient in QCS, exhibited its anti-inflammatory potential on UC (Yu et al., 2021).

The intracellular NOD-like receptor nucleotide-binding domain-like receptor family pyrin domain-containing-3 (NLRP3) is a critical controller of intestinal homeostasis by controlling various inflammatory and autoimmune conditions (Huang et al., 2020). The multi-protein complex of NLRP3, called NLRP3 “inflammasome,” has been related to plenty of inflammatory and autoimmune conditions such as UC (Zong et al., 2020). It is generally believed that these diseases are related to a genetic environment-mediated mucosal immune response disorder (Xavier and Podolsky, 2007; Pu et al., 2020). The NLRP3 inflammasome plays a primary role in the host’s defense of its role as a sensor for microbial and other danger signals (Zhao et al., 2019). A recent study has shown that the NLRP3 inflammasome is also a critical regulator of intestinal homeostasis (Huang et al., 2020). Activation of the NLRP3 inflammasome is generally believed to be associated with UC, suggesting that inhibiting the NLRP3 inflammasome activation may be an option to treat such inflammatory disorders (Zhao et al., 2019). It is reported that GA suppressed inflammation in the experimental colitis of rats and mice (Pandurangan et al., 2015; Li et al., 2019; Zhu et al., 2019; Shree et al., 2020). However, the mechanism of GA treating UC *via* inhibiting the NLRP3 inflammasome remains unclear. In this study, we focus on the effect of GA on experimental colitis induced by dextran sulfate sodium (DSS) in mice and its influence on the NLRP3 inflammasome.

## Methods and materials

### Materials

DSS (MW. 36000–50000) was bought from MP Biomedicals (USA). GA (Cat. No. S30153 98.5% for *in vivo*, B20851 ≥ 98% for *in vitro*) and 5-aminosalicylic acid (5-ASA, Cat. No. S30083) were purchased from Yuanye (Shanghai, China). Lipopolysaccharides (LPS, Cat. No. L2880) were bought from Sigma-Aldrich (Germany).

### Animals

Male BALB/c mice (SPF class, 6–8 weeks old, weighing 18–22 g) were provided by the Experimental Animal Center, Shanghai University of TCM. All the mice were housed in a temperature- and humidity-controlled environment (six mice per cage, 20°C ± 2°C, and 40%–60% humidity) with a 12-h light/dark cycle to acclimatize for one week with free access to food and water. All animal experiment protocols were approved by the Animal Research Welfare Council of Shanghai University of Traditional Chinese Medicine (Ethics No. PZ200120).

### Modeling and drug treatment

The mice were divided into Control group, Model group, DSS+5-ASA group, DSS+40 mg/kg GA group, DSS+80 mg/kg GA group, and DSS+120 mg/kg GA group. To generate colitis, 3.5% DSS was orally administered to the mice through drinking water for 7 days (except Control group). GA at 40 mg/kg, 80 mg/kg, 120 mg/kg, and 100 mg/kg and 5-ASA (dissolved in 0.5% carboxy-methylcellulose sodium) were orally administered to the mice from day 7 to day 12. 5-ASA acted as the positive control. On day 13, *via* ether anesthesia, all mice were euthanized to collect blood samples and colon tissues. The blood samples of the mice were collected by removing the eyeball (Li et al., 2020).

### Histological evaluation

The tissues were fixed in 4% paraformaldehyde for 24 h, cut into 5-µm sections after dehydration, and embedded in paraffin. HE reagents were used to stain the colonic sections. Histological scores were evaluated by assessing the indices for changes in the epithelium and for cell infiltration (Dohi et al., 2005; Yu et al., 2021). The following were observed: 1) no notable signs of inflammation; 2) very low levels of leukocyte infiltration; 3) low levels of leukocyte infiltration; 4) high levels of leukocyte infiltration, moderate vascular density, and thickening of the colon wall; and 5) transmural infiltration, loss of goblet cells, severe vascular density, and thickening of the colon wall. Tissue sections were visualized and photographed under a microscope using a camera system (Nikon DS-U3, Japan).

### ELISA analysis

Colonic tissue samples and sera were collected from BALB/c mice. The expressions of TNF-α, IL-33, interleukin-18 (IL-18), IL-1β, and IFN-γ in serum and in the lysis buffer of the colon tissue were determined by ELISA (Merck, Germany) according to the manufacturer’s instructions.

### Real-time PCR

The protocols and the reagents of RT-qPCR have been described in detail previously (Yu et al., 2021). Total RNA was obtained from colon tissues using a TRIzol reagent (Biotime, China) following the manufacturer’s instructions. After RNA concentration was determined, complementary DNA (cDNA) was generated using an RT-PCR reagent (Thermo Fisher Scientific Inc., Vilnius, Lithuania). Next, RT-PCR was conducted using the FastStart Universal SYBR Green Master (Rox) (Roche, Basel, Switzerland) through a LightCycler 96 q-PCR system (Roche, Basel, Switzerland). The glyceraldehyde 3-phosphate dehydrogenase (GAPDH) acted as a control for the total mRNA amount. The results were detected using the 2^−△△CT^ method. The PCR primer sequences in this work are shown in Table 1.

**TABLE 1:** Primer used for RT-qPCR.

Gene	Sequence	Produce length
NLRP3	Forward: GGA​AGA​TTA​CCC​GCC​CGA​GA	273
	Backward: CCC​AGC​AAA​CCC​ATC​CAC​TC	
Caspase-1	Forward: TGT​ATT​CAC​GCC​CTG​TTG​GAA	115
	Backward: TTG​CTT​CCT​CTT​TGC​CCT​CA	
Caspase-4	Forward: TGG​CTG​AAA​ACA​AAC​ACC​CTG	158
	Backward: CTT​GTC​ACT​GCG​TTC​AGC​AT	
IL-1β	Forward: TGC​CAC​CTT​TTG​ACA​GTG​ATG	220
	Backward: AAG​GTC​CAC​GGG​AAA​GAC​AC	
IL-18	Forward: CTT​TGA​GGC​ATC​CAG​GAC​AAA	154
	Backward: CAC​AGG​GGA​GAA​GTG​AAA​GCA	
ASC	Forward: GAC​AGT​ACC​AGG​CAG​TTC​GT	96
	Backward: AGT​CCT​TGC​AGG​TCA​GGT​TC	
GAPDH	Forward: ACT​TTG​GCA​TTG​TGG​AAG​GG	225
	Backward: CGG​ACA​CAT​TGG​GGG​TAG​GA	

### Immunohistochemistry

Immunohistochemistry (IHC) was performed following previously described methods (Yu et al., 2021). The colonic tissue was fixed in 4% neutral formalin, dehydrated with increasing concentrations of ethanol and dimethyl benzene, embedded in paraffin, and cut into 5-μm sections. The paraffin section was mounted on slides, cleaned, and hydrated. The section was treated with a buffered blocking solution (3% bovine serum albumin in PBS) for 25 min. Then, the sections were incubated with a primary antibody against NLRP3 (1:100) at 4°C for 24 h and incubated with a secondary antibody at room temperature for 50 min. The reaction was blocked by washing with PBS for 5 min. Images were acquired through a microscope using a camera system (Nikon DS-U3, Japan).

### Cell culture

RAW264.7 cell lines were bought from the Chinese Academy of Sciences (Shanghai, China). Dulbecco’s modified Eagle medium (DMEM, Sigma, United States) supplemented with penicillin (100 U/mL), streptomycin (100 μg/mL), and 10% fetal bovine serum (FBS, Sigma, United States) was used for culture at 37°C with 5% CO_2_ in an incubator. Cell lines were collected until the logarithmic growth phase for the experiment.

### Cytotoxicity assay

For the toxicology experiment, RAW264.7 cell lines were seeded onto a 96-well plate at 5 × 10^3^ cells per well and cultured overnight. Then RAW264.7 cells were incubated with serial concentrations of GA dissolved in 0.1% dimethylsulfoxide (DMSO) for 24 h. The concentrations of GA were 12.5 μM, 25 μM, 50 μM, 100 μM, and 200 μM. Next, 10 μL of Cell Counting Kit-8 (CCK-8, Dojindo, Japan) solution was added to each well. The culture plate was placed in the incubator for 2 h, and the absorbance of the viable cells was detected (450 nm) using a Multiskan FC microplate reader (Thermo Scientific, United States).

### Nitric oxide production determination

RAW264.7 cells were inoculated into 6-well plates for 24 h and then treated with 50 μM GA, 100 μM GA, and 200 μM GA and 1 μg/mL LPS for 24 h. The amounts of NO release were determined by a nitric oxide assay kit (Beyotime, China). In brief, 50 μL standard and cell supernatants were added to the 96-well plate and mixed with 50 μL Griess Reagent I and 50 μL Griess Reagent II at room temperature, respectively. The OD value was examined by a microplate reader at 562 nm.

### Western blotting

Total protein was extracted by cell samples and colonic tissue of different treatment groups in the lysis buffer. The cells were collected and incubated in an RIPA buffer (with the addition of phosphorylation inhibitor and protease inhibitor) for 20 min in an ice bath. After centrifugation at 15,000 rpm for 15 min at 4°C, the upper-clarified lysate was collected. For the colon tissue, 700 μL RIPA lysate was added to a 100-mg sample to extract the protein. Protein concentration was determined using a BCA kit (Beyotime Biotechnology).

For western blotting, the extracted protein of each group was separated by 8%–12% SDS-PAGE and transferred onto a polyvinylidene fluoride membrane. The membrane was blocked with a 5% BSA solution. Then, the membrane was cut into different blots and probed with specific primary antibodies (Table 2) overnight at 4°C (12 h). Next, the membrane was washed with 1 × PBST and incubated with secondary antibodies (Beyotime Biotechnology) at room temperature for 1 h. Then, it was incubated with an enhanced chemiluminescent substrate (Beyotime Biotechnology). In this experiment, β-actin, α-tubulin, HSP90, and GAPDH were used as an internal control to confirm that the amounts of loaded protein were equal. All experiments were repeated at least three times. Western blotting plots were quantified using ImageJ software (United States).

**TABLE 2:** Primary antibody list.

Primary antibody	Company	Dilution
Caspase-1	Millipore	1:1000
Cleaved Caspase-1	CST	1:500
NLRP3	Abcam	1:1000
α-tubulin	Beyotime	1:1000
GAPDH	Millipore	1:1000
Cleaved IL-1β	CST	1:500
IL-1β	Millipore	1:1000
iNOS	Abcam	1:1000
COX2	Abcam	1:1000
p65-NF-κB	Abcam	1:1000
P-p65-NF-κB	Abcam	1:1000
ERK	Abcam	1:1000
P-ERK	Abcam	1:1000
JNK	Abcam	1:1000
P-JNK	CST	1:2000
p38	CST	1:1000
P-p38	CST	1:1000
HSP90	Abcam	1:1000

### Statistical analysis

All experiments were performed at least three times, and the results were expressed as mean ± SD. The gray value of the protein was quantified by ImageJ software. Data were analyzed using the software SPSS version 25.0 and illustrated using Graphpad Prism 8.0. The differences between the two groups were analyzed with *t*-test. ANOVA was performed for multiple comparisons. *p* < 0.05 was considered statistically significant.

## Results

### GA relieved murine colitis

**FIGURE 1:**
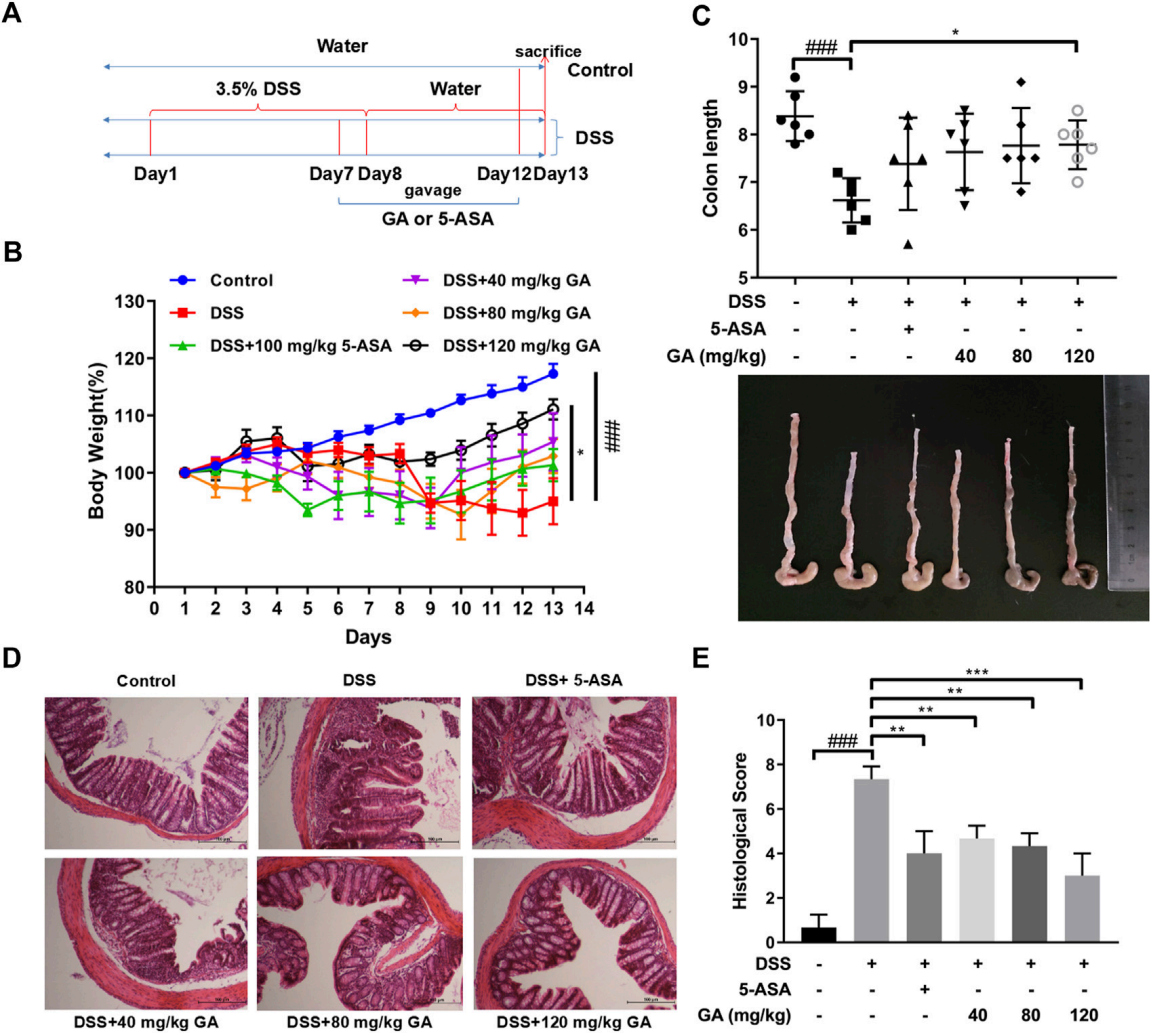
GA attenuated DSS-induced murine colitis. BALB/C mice were orally administrated with GA 40 mg/kg, 80 mg/kg, and 120 mg/kg once daily from day 7 to day 12. 5-ASA was orally administrated with 100 mg/kg for the positive Control group. **(A)** Experiment design. **(B)** Body weights were measured daily from day 1 to day 13 and performed as a ratio to that at day 1. **(C)** Colon length. **(D)** Colon sections (100×) were examined using HE staining. **(E)** Histological score. Data are presented as mean ± SD (n = 6); ###*p* < 0.001 vs. Control group by *t*-test; *p < 0.05, **p < 0.01, and ***p < 0.001 vs. DSS group.

The experiments were designed and carried out as described in the section entitled “*Methods and materials*” (Figure 1A). Compared with the Control group, a severe weight loss of mice was observed in the DSS group (*p* < 0.001). GA treatment at 120 mg/kg attenuated DSS-induced weight loss (*p* < 0.05) (Figure 1B). Similarly, DSS typically caused colonic shortening (*p* < 0.001), while 120 mg/kg GA ameliorated this change (*p* < 0.05) (Figure 1C). HE staining of colon sections in the DSS group showed an enormous inflammatory cell infiltration with epithelial cell damage, edematous, hemorrhagic, and ulcerated groups compared with the Control group. 5-ASA significantly alleviated these symptoms induced by DSS (*p* < 0.01), while GA (40 mg/kg, 80 mg/kg, and 120 mg/kg) restored colon damage, improved crypt architecture, relived congestion and edema (Figure 1D), and reduced the histological scores remarkably (*p* < 0.01) (Figure 1E).

### GA regulated the cytokine expression in UC mice

**FIGURE 2:**
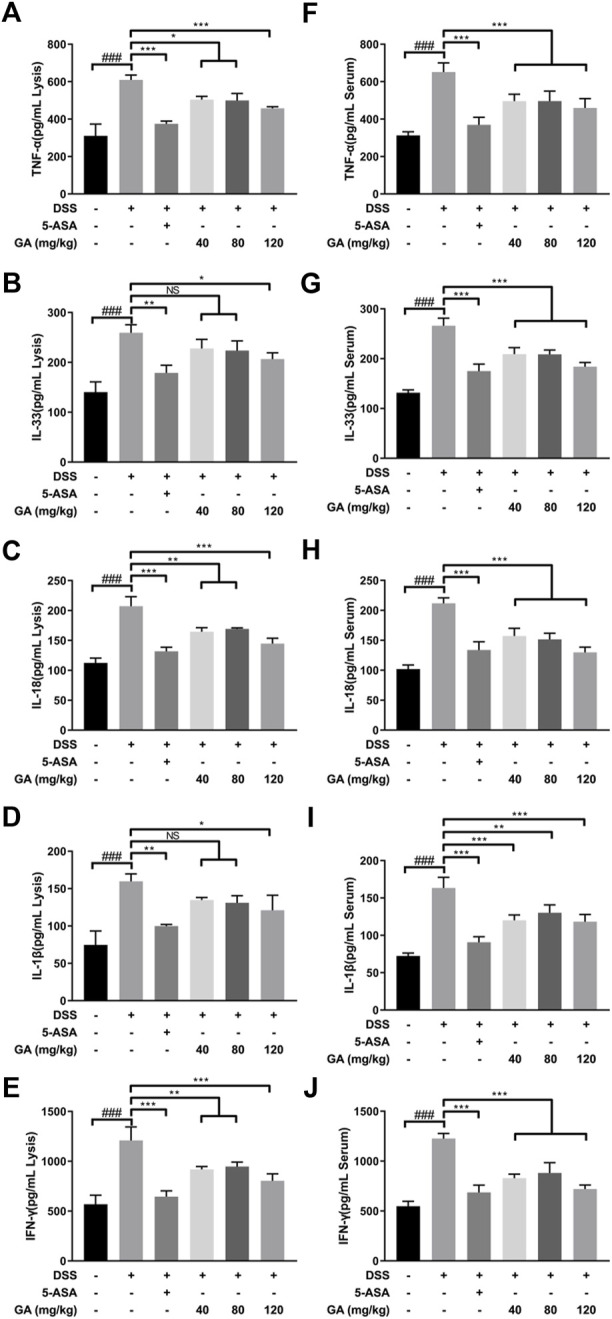
GA regulated the cytokine expression in the colon tissue of mice with DSS-induced colitis. The contents of **(A)** TNF-α, **(B)** IL-33, **(C)** IL-18, **(D)** IL-1β, and **(E)** IFN-γ in the colon tissue and contents of **(F)** TNF-α, **(G)** IL-33, **(H)**IL-18 **(I)**, IL-1β, and **(J)** IFN-γ in the serum of mice with DSS-induced colitis were determined using an ELISA kit. Data are mean ± SD. (n ≥ 3). ###*p* < 0.001 vs. Control group by *t*-test; *p < 0.05, **p < 0.01, and ***p < 0.001 vs. DSS group.

The secretions of TNF-α, IL-33, IL-18, IL-1β, and IFN-γ in both the serum and colon tissues of mice in the DSS group were significantly upregulated compared with the Control group (Figure 2), indicating DSS-induced inflammation. Compared with the DSS group, 5-ASA and GA markedly inhibited the secretions of TNF-α, IL-33, IL-18, IL-1β, and IFN-γ in both the serum and colon tissues.

### GA regulated inflammasome mRNA expression *in vivo*

**FIGURE 3:**
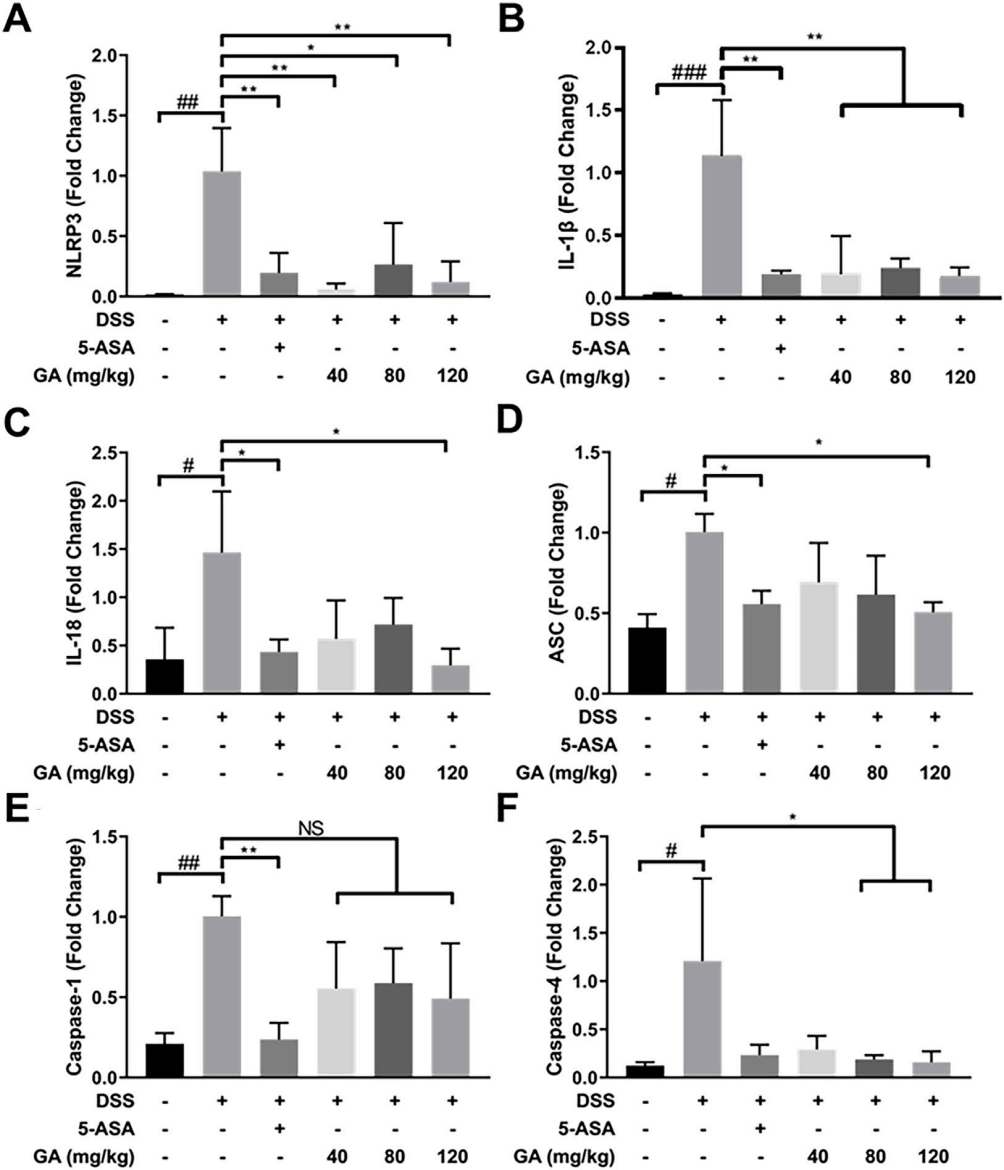
GA regulated the inflammasome mRNA expression in the colon tissue of mice with DSS-induced colitis. RNA of each group was extracted from the colon tissue and converted to cDNA. The expressions of **(A)** NLRP3, **(B)** IL-1β, **(C)** IL-18, **(D)** ASC, **(E)** Caspase-1, and **(F)** Caspase-4 were determined by RT-qPCR. Data are presented as mean ± SD (n = 3). #*p* < 0.05, ##*p* < 0.01, ###*p* < 0.001 vs. Control group by *t*-test; *p < 0.05, **p < 0.01, and ***p < 0.001 vs. DSS group.

DSS-induced murine colitis showed many pathological features on UC in humans, especially type I inflammatory response. Compared with the Control group, the mRNA expression of NLRP3, ASC, Caspase-1, Caspase-4, IL-1β, and IL-18 significantly increased in the DSS group (Figure 3). 5-ASA and GA dramatically lowered the upregulated mRNA expressions of these inflammasomes induced by DSS (Figure 3), indicating 5-ASA and GA were effective.

### GA suppressed NLRP3 expression to inhibit inflammasome activation

**FIGURE 4:**
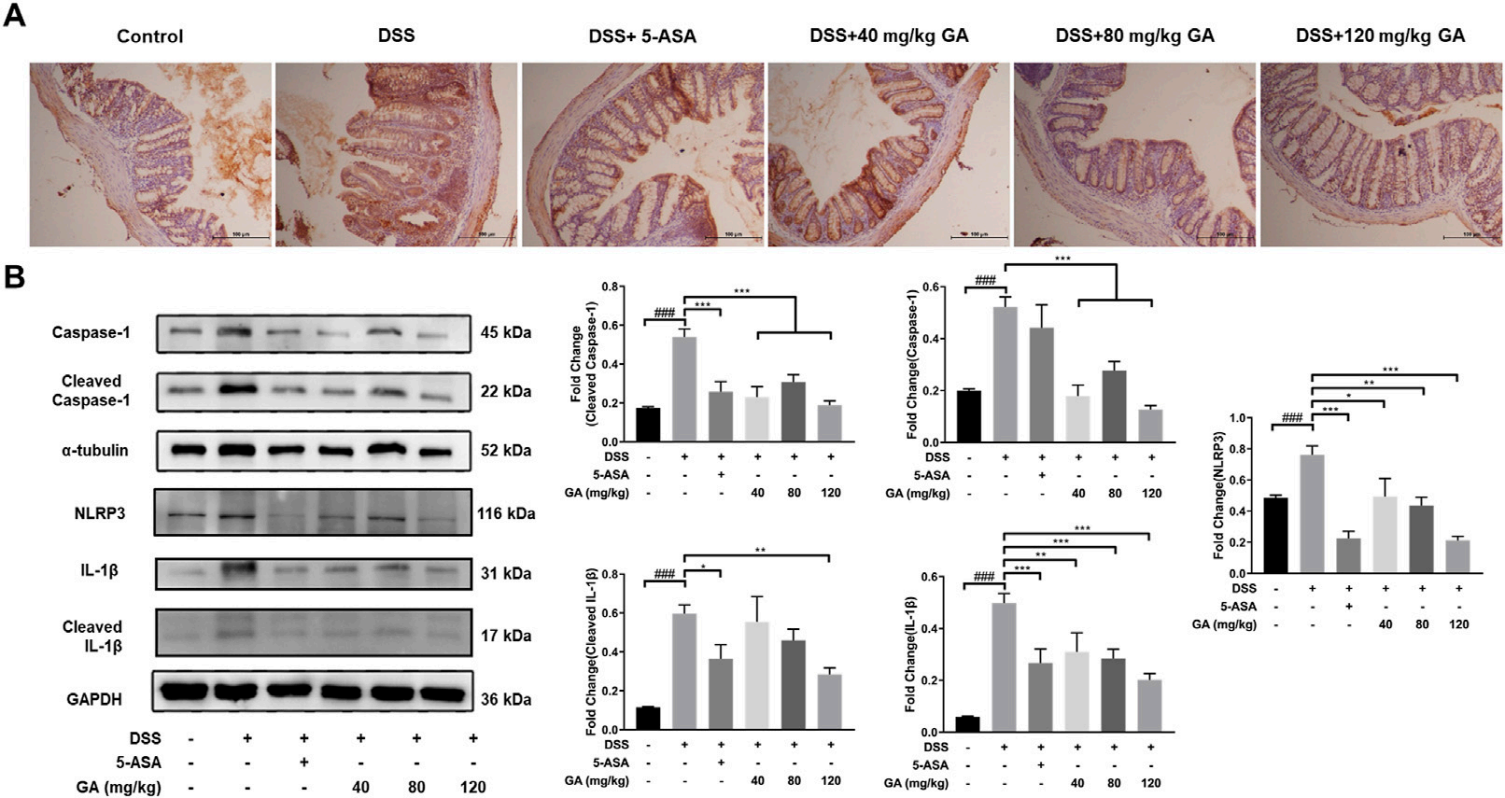
GA suppressed NLRP3 expression to inhibit inflammasome activation. **(A)** Immunohistochemistry results (100×) of NLRP3 staining in the colon tissue of mice with DSS-induced colitis. **(B)** Total protein was extracted from the colon tissue of different groups of mice. Protein expressions of Caspase-1, cleaved Caspase-1, NLRP3, IL-1β, and cleaved IL-1β in the colon tissue were measured using western blotting. Data are presented as mean ± SD (n = 3). ##*p* < 0.01, ###*p* < 0.001 vs. Control group by *t*-test; *p < 0.05, **p < 0.01, and ***p < 0.001 vs. DSS group.

We have demonstrated that GA reduced the levels of TNF-α, IL-33, IL-18, IL-1β, and IFN-γ in the serum and colon tissues, which provides the possibility of suppressing the activation of NLRP3 inflammasomes. We also detected a relative downstream protein expression of NLRP3. IHC results illustrated that the expressions of NLRP3 were hardly found in the Control group but were significantly elevated in the DSS group, and after treatment of GA, the expression of NLRP3 was reduced (Figure 4A). The results of western blotting revealed that GA treatment notably repressed the over-expression of NLRP3 and Caspase-1 induced by DSS, leading to IL-1β secretion reduction (Figure 4B). Those results indicated that GA might have an anti-inflammatory ability *via* inhibiting the activation of the NLRP3 inflammasome.

### GA suppressed inflammatory response stimulated by LPS in RAW264.7 macrophages

**FIGURE 5:**
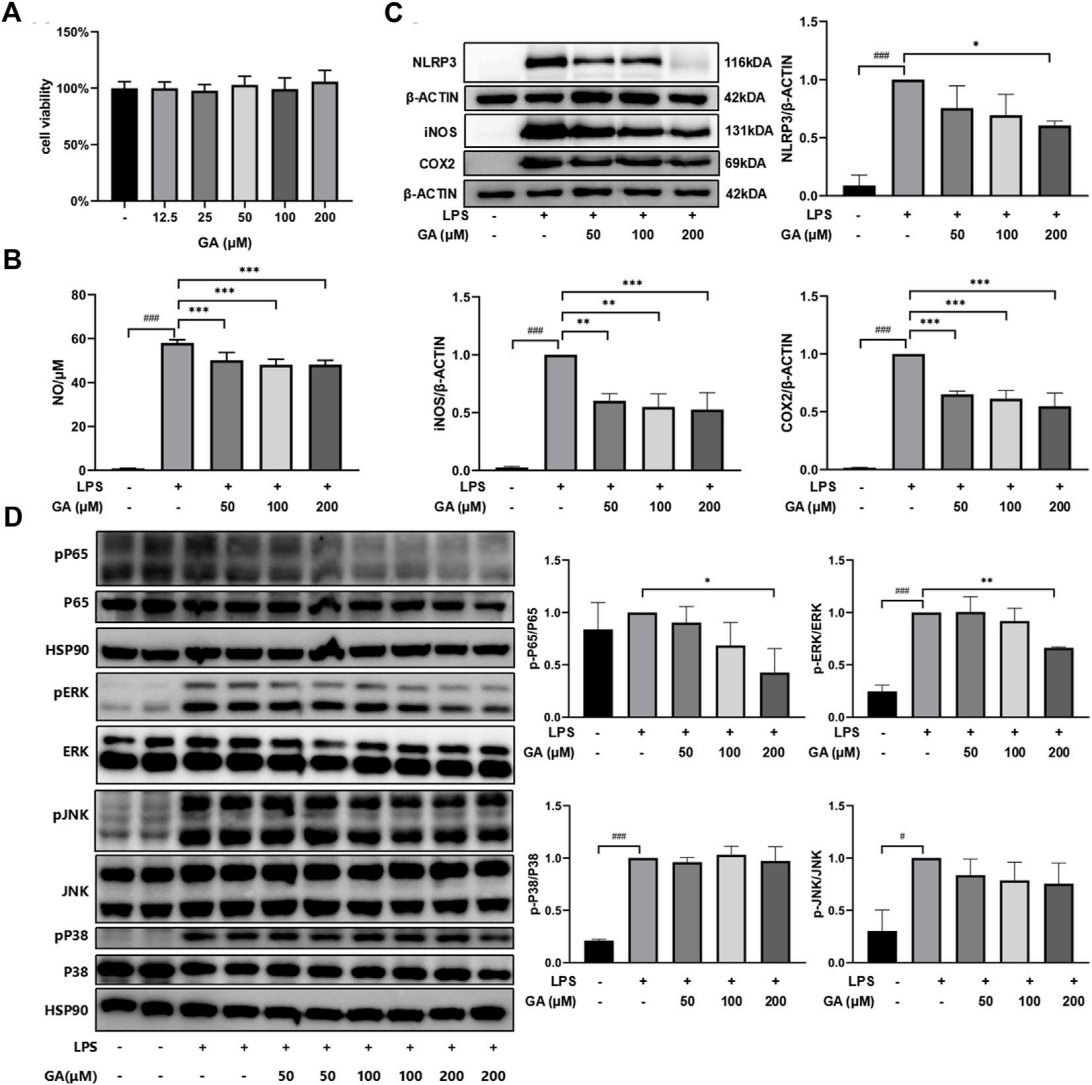
GA suppressed inflammatory response in LPS-stimulated RAW264.7 macrophages. Cells were co-treated with GA at 50, 100, and 200 μM in the presence of LPS (1 μg/mL) for 24 h. **(A)** Cell viability was measured by using CCK-8 assay. **(B)** NO production was determined by Griess reagents. **(C)** The protein expressions of NLRP3, iNOS, and COX2 in LPS-stimulated RAW264.7 cells were measured using western blotting. **(D)** The protein expressions of p65-NF-κB, ERK, JNK, and P38 in the LPS-stimulated RAW264.7 cell were measured in western blotting. Data are presented as mean ± SD (n = 3). ^###^ *p* < 0.001 vs. Control group by *t*-test; *p < 0.05, **p < 0.01, and ***p < 0.001 vs. LPS group.

The CCK-8 assay indicated that the concentrations of GA applied in the subsequent experiments exhibited no evident influence on RAW264.7 cell viability (Figure 5A). NO release was remarkably increased in the presence of LPS compared with untreated cells (*p* < 0.001) and was suppressed after GA treatment (Figure 5B). Similarly, the protein expressions of NLRP3, iNOS, and COX2 were dramatically upregulated with LPS stimulation (Figure 5C). GA treatment significantly lowered the LPS-stimulated elevation of NLRP3 at 200 μM (*p* < 0.05) and dose-dependently downregulated LPS-induced iNOS and COX2 expressions (*p* < 0.01). Further investigation demonstrated that GA significantly reduced the phosphorylation of p65-NF-κB (*p* < 0.05) and ERK (*p* < 0.01) in the RAW264.7 cell primed by LPS (Figure 5D).

## Discussion

UC is an inflammatory disease of the intestine, highly susceptible to relapse and progression into cancer (Ordas et al., 2012). Although the pathogenesis of UC is unclear in modern medicine, it is commonly regarded to be associated with immune inflammation (Zhou et al., 2021).

Inflammasomes play a critical role in the progression of UC (Yu et al., 2021). The inflammasomes are cytoplasmic complexes that can recognize pathogen-associated molecular patterns and damage-associated molecular patterns (Neumann et al., 2018; Pourcet et al., 2018). When stimulated, the components of the inflammasomes combine to form complexes, which subsequently produce and release large amounts of pro-inflammatory cytokines through a series of enzymatic reactions (Broz and Dixit, 2016), whereas the NLRP3 inflammasome is one of the most characteristic (Shao et al., 2015). The NLRP3 inflammasome is composed of three main components: the sensor NLRP3 protein, the adaptor ASC, and the effector protein, Caspase-1 (Lazaridis et al., 2017; Montoya et al., 2018). When the host receives exogenous or endogenous stimuli, NLRP3 will be activated and recruit ASC and Caspase-1. The stimulated NLRP3 interplays with ASC, and pro-Caspase-1 combines with ASC into a cytoplasmic complex, triggering Caspase-1’s activation (Wang et al., 2016; Qu et al., 2019). The active Caspase-1 then cleaves IL-18 and IL-1β from their prototypes to biologically active forms (Furuya et al., 2018; Lopes de Oliveira et al., 2019). These cytokines participate in inducing inflammation (He et al., 2015). Clinical trials showed that NLRP3 was involved in the inflammatory response in many diseases. The NLRP3 inflammasome was activated in UC patients and was associated with the severity of UC (Chen et al., 2020). It was also demonstrated that in the DSS-induced mouse model, the NLRP3 inflammasome was activated and released large amounts of pro-inflammatory cytokines, IL-1β and IL-18, causing inflammatory damage to colonic mucosal tissue. Inhibition or knockdown of the NLRP3 inflammasome could alleviate the severity of DSS-induced colitis, control the release of IL-1β and IL-18, and suppress the inflammatory response of UC (Kelley et al., 2019; Lv et al., 2021). Thus, the NLRP3 inflammasome plays a crucial part in the pathogenesis of colitis, and the control of NLRP3 inflammasome activation could improve the symptoms of UC.

In our previous study, we found that QCS ameliorated DSS-induced colitis by inhibiting the expression of Caspase-1 and IL-1β during inflammasome activation (Yu et al., 2021). GA, the main effective compound in QCS (Zhou et al., 2021), is a very safe phenolic acid with good anti-inflammatory effects. It has been reported that long-term consumption of GA has no adverse effects on health conditions, which reflects GA’s high safety levels (Yang et al., 2022). It has benn documented that GA prevented 1,2-dimethylhydrazine-induced colon inflammation and attenuated DSS-induced colitis in rats (Li et al., 2019; Shree et al., 2020). In addition, GA suppressed inflammation in both DSS- and TNBS-induced UC of BALB/c mice *via* inhibiting the NF-κB pathway (Pandurangan et al., 2015; Zhu et al., 2019). Consistent with these observations, our data showed that GA attenuated weight loss, relieved symptoms, and ameliorated colonic morphological injuries of BALB/c mice, induced by DSS (Figure 1). Furthermore, the present study demonstrates that GA lowered the contents of IL-1β and IL-18 in serum and colon tissues of BALB/c mice, elevated by DSS (Figure 2), and downregulated the protein and mRNA expressions of the NLRP3 pathway in the colon tissue (Figures 3, 4), demonstrating that GA inhibited the activation of the NLRP3 inflammasome. Immunohistochemistry was used to detect the expression of NLRP3 in colonic tissues of mice with DSS-induced colitis. The results indicated that GA significantly downregulated the NLRP3 expression, consistent with its inflammatory intervention in DSS-induced colitis (Figure 4). According to other evidence, GA suppressed the NLRP3 inflammasome activation in gouty arthritis of C57BL/6J mice and LPS-primed murine J774A.1 cell and bone marrow-derived macrophages (Lin et al., 2020). Moreover, the NLRP3 inflammasome pathway has been proposed to be involved in the maturation of IL-33 (Neumann et al., 2018), and IL-18 can induce the production of IFN-γ (Lv et al., 2021). Our findings also demonstrate that GA reduced the production of various cytokines including IL-33, TNF-α, and IFN-y in the serum and colon tissues, elevated by DSS (Figure 2).

**FIGURE 6:**
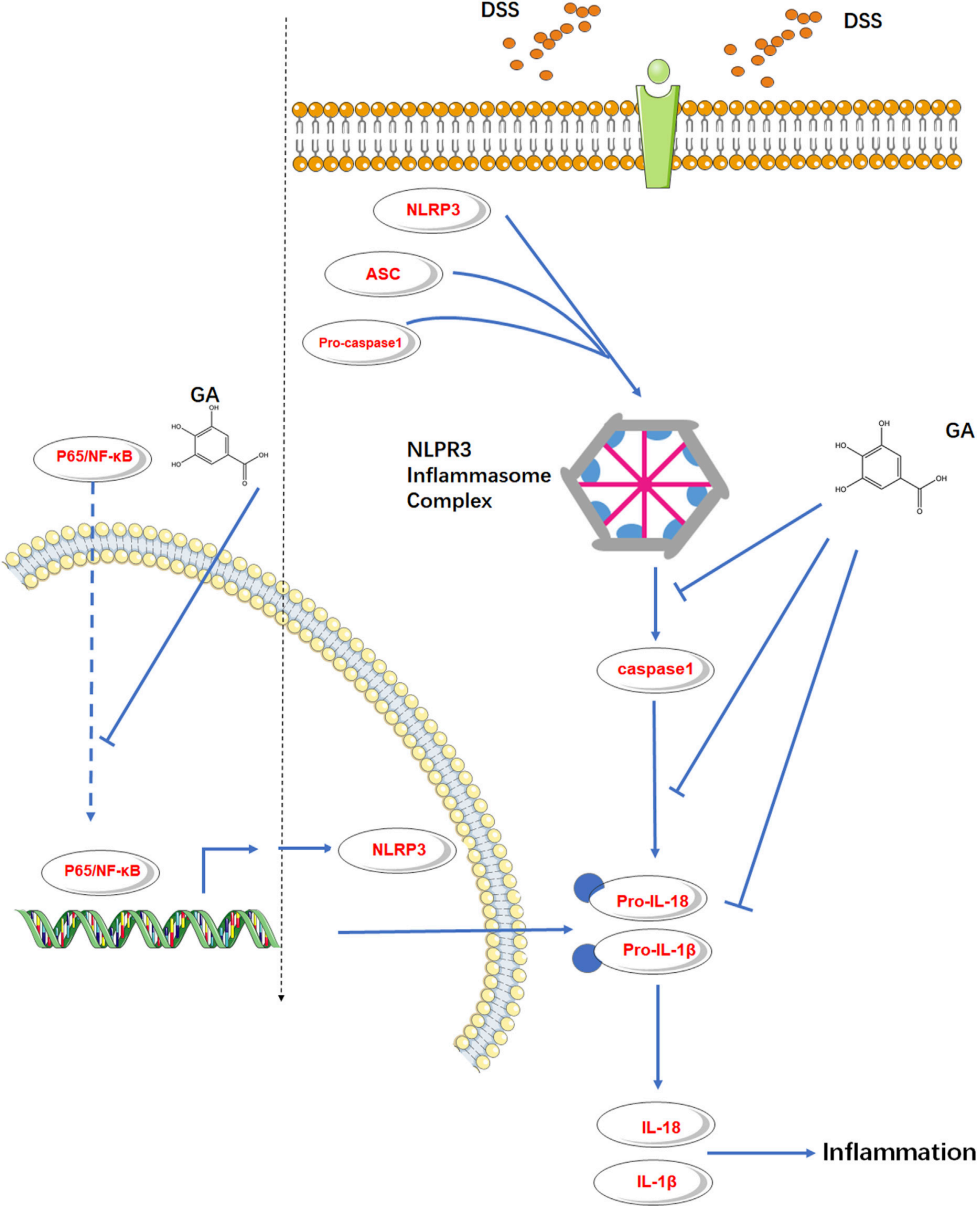
GA inhibited the endogenous NLRP3 expression and NLRP3 inflammasome activation in murine colitis.

It is generally believed that NLRP3 must be primed before activation and formation of the inflammasome complex. An NF-κB-activating stimulus such as LPS priming first induces elevated expressions of NLRP3 and IL-1β (Guo et al., 2015). Murine RAW264.7 macrophage is naturally deficient in ASC adaptor protein (Pelegrin et al., 2008; He et al., 2015), but it still expresses endogenous NLRP3 (Chen and Chen, 2018). Pandurangan et al. (2015) illustrated that GA inhibited the p65-NF-κB protein expression in LPS-induced RAW264.7 cells. Therefore, we used the RAW264.7 cell induced by LPS to probe the effect of GA on the downstream protein of p65-NF-κB, NLRP3. The *in vitro* results verified our speculation that GA not only inhibited NLRP3 inflammasome activation but also inhibited the endogenous NLRP3 expression subsequently with a suppression of p65-NF-κB phosphorylation (Figure 6).

In conclusion, this study shows that GA ameliorated DSS-induced UC in mice *via* inhibiting the NLRP3 inflammasome. Herbs and preparations containing GA may have an anti-inflammatory effect on UC, but the deeper mechanisms deserve further investigation.

## Data Availability

The original contributions presented in the study are included in the article/Supplementary Materials; further inquiries can be directed to the corresponding author.

## Abbreviations

5-ASA, 5-aminosalicylic acid; ANOVA, analysis of variance; ASC, apoptosis-associated speck-like protein containing a CARD; CECs, colonic epithelial cells; DSS, dextran sodium sulfate; ELISA, enzyme-linked immunosorbent assay; HBSS, Hank’s balanced salt solution; HE, hematoxylin and eosin; IBD, inflammatory bowel disease; IL-1β, Interleukin 1β; LPS, lipopolysaccharide; NF-κβ, nuclear factor kappa-β; NLRP3, nucleotide-binding domain-like receptor family pyrin domain containing 3; NO, nitric oxide; PBS, phosphate-buffered saline; QCS, Qingchang Suppository; UC, ulcerative colitis.
